# Capturing the Kidney Transcriptome by Urinary Extracellular Vesicles—From Pre-Analytical Obstacles to Biomarker Research

**DOI:** 10.3390/genes14071415

**Published:** 2023-07-08

**Authors:** Karina Barreiro, Om Prakash Dwivedi, Antti Rannikko, Harry Holthöfer, Tiinamaija Tuomi, Per-Henrik Groop, Maija Puhka

**Affiliations:** 1 Institute for Molecular Medicine Finland FIMM, HiLIFE, University of Helsinki, 00290 Helsinki, Finland; karina.barreiro@helsinki.fi (K.B.); om.dwivedi@helsinki.fi (O.P.D.); harry.holthofer@helsinki.fi (H.H.); tiinamaija.tuomi@hus.fi (T.T.); 2 Institute for Molecular Medicine Finland FIMM, EV and HiPREP Core, University of Helsinki, 00290 Helsinki, Finland; 3 Research Program in Systems Oncology, Faculty of Medicine, University of Helsinki, 00290 Helsinki, Finland; antti.rannikko@hus.fi; 4 Department of Urology, University of Helsinki, Helsinki University Hospital, 00290 Helsinki, Finland; 5 III Department of Medicine, University Medical Center Hamburg-Eppendorf, 20246 Hamburg, Germany; 6 Lund University Diabetes Centre, Department of Clinical Sciences, Lund University, 214 28 Malmö, Sweden; 7 Folkhälsan Institute of Genetics, Folkhälsan Research Center, 00290 Helsinki, Finland; per-henrik.groop@helsinki.fi; 8 Endocrinology, Abdominal Centre, Helsinki University Hospital, 00029 Helsinki, Finland; 9 Department of Nephrology, University of Helsinki, Helsinki University Hospital, 00290 Helsinki, Finland; 10 Research Program for Clinical and Molecular Metabolism, Faculty of Medicine, University of Helsinki, 00290 Helsinki, Finland; 11 Department of Diabetes, Central Clinical School, Monash University, Melbourne, VIC 3800, Australia

**Keywords:** urinary extracellular vesicles, exosomes, urine, diabetic kidney disease, reference genes, miRNA, mRNA, sequencing

## Abstract

Urinary extracellular vesicles (uEV) hold non-invasive RNA biomarkers for genitourinary tract diseases. However, missing knowledge about reference genes and effects of preanalytical choices hinder biomarker studies. We aimed to assess how preanalytical variables (urine storage temperature, isolation workflow) affect diabetic kidney disease (DKD)—linked miRNAs or kidney—linked miRNAs and mRNAs (kidney-RNAs) in uEV isolates and to discover stable reference mRNAs across diverse uEV datasets. We studied nine raw and normalized sequencing datasets including healthy controls and individuals with prostate cancer or type 1 diabetes with or without albuminuria. We focused on kidney-RNAs reviewing literature for DKD-linked miRNAs from kidney tissue, cell culture and uEV/urine experiments. RNAs were analyzed by expression heatmaps, hierarchical clustering and selecting stable mRNAs with normalized counts (>200) and minimal coefficient of variation. Kidney-RNAs were decreased after urine storage at −20 °C vs. −80 °C. Isolation workflows captured kidney-RNAs with different efficiencies. Ultracentrifugation captured DKD -linked miRNAs that separated healthy and diabetic macroalbuminuria groups. Eleven mRNAs were stably expressed across the datasets. Hence, pre-analytical choices had variable effects on kidney-RNAs—analyzing kidney-RNAs complemented global correlation, which could fade differences in some relevant RNAs. Replicating prior DKD-marker results and discovery of candidate reference mRNAs encourages further uEV biomarker studies.

## 1. Introduction

Extracellular vesicles (EV) are nowadays a hot topic in the biomarker research field [1,2,3,4]. Urinary EV (uEV) are of particular interest for pathologies of the genitourinary tract [5,6,7,8]. Specifically for diabetic kidney disease (DKD), a microvascular complication of diabetes, uEV are a promising source of non-invasive biomarkers [9,10,11] that might complement, reduce the need for or eventually even replace kidney biopsies and facilitate early diagnostics and prognosis.

Currently, effort is put on research and to set forth recommendations for uEV work, e.g., in sample handling, storage, uEV isolation and reporting [12,13,14,15,16,17,18]. This is highly important because there are vast differences in the pre-analytical, analytical and reporting procedures. For example, a recent survey by the Spanish Society for Research and Innovation (Spain) in Extracellular Vesicles (GEIVEX) found that the variability of preanalytical procedures can be as high as 94% [18]. Without some level of standardization, the biomarker discovery results are seldom highly robust or reproducible [19].

More specifically, one of the most pressing problem in the preanalytical part is that many collections in laboratories and biobanks may not be handled and stored optimally for uEV research. Moreover, only few studies have characterized the effect of pre-analytical variables on the uEV, especially regarding the end-point biomolecular level used in biomarker studies, e.g., the transcriptome [14]. Equally, only a few studies have comprehensively characterized the effect of EV isolation methods on transcriptomics [13,15,16,20,21,22]. Thus, it is difficult to compare results between dissimilar studies.

Urinary EV capture kidney transcriptome and proteome ([7,9,23,24,25,26]. With focus on uEV RNAs, we and others have shown that uEV can capture the dysregulation of RNAs associated with pathological mechanisms of DKD such as oxidative stress [9], fibrosis [27], and inflammation [28]. We have previously shown by RNA sequencing technologies that some preanalytical variables such as urine storage temperature and isolation methods affect the uEV RNA yield and global miRNA and mRNA profiles [9,14]. However, for kidney research, it would be important to understand how exactly the pre-analytical choices affect the uEV as a “liquid kidney biopsy”. Are all the uEV miRNAs and mRNAs—highly or specifically expressed by the kidney and from different disease mechanism pathways—affected by the different preanalytical variables and to which extent? Are the kidney derived RNAs for example missing completely or just downregulated and therefore still available as biomarkers?

Urinary EV reference genes represent another unmet need in the EV field. Both research on EV reference genes and recommendations on how to select the reference genes are increasing [29,30]. However, only a moderate number of sequencing datasets are currently available for rigorous search of robust reference genes that would be stable across studies, at least for uEV. Again, the effect of preanalytical variables, or demographic and disease status variables, on the stability of reference genes is not clear. This represents a problem for qPCR validation experiments. GAPDH is commonly utilized to normalize gene expression but does not work equally fine for all tissues, biofluids, or disease status [31,32,33]. In conclusion, it is unclear how candidate markers reported by different studies could be replicated under different experimental conditions.

In this study, we assessed the effect of storage temperature and uEV isolation workflows on uEV transcriptome by focusing on highly expressed miRNAs and enriched genes of the kidney. We assessed the replicability of previously described candidate miRNA markers of DKD and explored the existence of reference genes across diverse uEV sequencing datasets.

## 2. Methods

### 2.1. miRNA and mRNA Sequencing Datasets

The datasets included in this study were retrieved from previous publications from our group describing the pre-analytical and analytical parts including quality control in detail [6,9,13,14]. Details for each dataset are included in Table 1. For the storage temperature study, urine samples were divided in two aliquots on the collection day, and they were stored at −20 °C or −80 °C for 13–16 months. Importantly, temperature sample pairs were always stored for equal times i.e., the isolation of extracellular vesicles was done the same day. Similarly, there were no differences in the storage time for isolation workflow, overnight (ON)/24 h collections (24 h), and pre-clearing studies between the sample pairs. Of note, except for the isolation workflow dataset, the rest of the samples were processed by ultracentrifugation.

### 2.2. Kidney Top Expressed miRNAs and Kidney Enriched mRNAs in uEV

Kidney enriched genes (“At least four-fold higher mRNA level in kidney compared to the average level in all other tissues”) were retrieved from The Human Protein Atlas, v20 [34,35] (www.proteinatlas.org) (accessed on 19 November 2020). For miRNAs, we used top kidney expressed miRNAs (40 miRNAs with highest expression in the kidney) which were retrieved from miRNATissueAtlas2 [36] (https://ccb-web.cs.uni-saarland.de/tissueatlas2) (accessed on 17 June 2022). For these analysis, raw sequencing counts were normalized as described in the original publications by using TMM (trimmed mean of M values) [37] in edgeR [38] or DEseq2 normalization [39,40].

### 2.3. Literature Review of miRNAs Associated with DKD

We did a literature review of miRNAs associated with DKD based on evidence from tissue (human or animal models) or in vitro models and for miRNAs based on evidence from human urine, urinary sediments or uEV (differential expression padj < 0.05). For the latter, some studies reported miRNAs with nominal *p*-values, and in such cases we included only the miRNAs that had been also validated with another quantification method or by using in-vitro or in-vivo models. To search the DKD associated miRNAs in our uEV dataset we used the reported identifiers i.e., if the literature only provided the stem identifier, we searched the immature miRNA in our dataset and not the mature miRNAs (-3p/-5p).

### 2.4. Stable mRNAs across Datasets

All datasets were normalized using TMM normalization. Of note, samples from overnight and 24 h collections, with and without pre-clearing and technical replicas were normalized together and we refer to this dataset as “technical dataset”. Genes with normalized counts of CPM > 200 in all samples were filtered to calculate the coefficient of variation (CV). The top 100 genes with the lowest CVs were selected from each experimental dataset and the gene lists were compared to identify shared genes across datasets. To assess the stable genes functions we used gene cards [41] (www.genecards.org) (accessed on 27 June 2023) and to assess to which pathways the stable genes contribute to, we used Uniprot knowledge base (UniProt Consortium 2023) (https://www.uniprot.org/) (accessed on 28 April 2023). Protein interaction was assessed using STRING V11.5 [42] (https://string-db.org/) (accessed on 28 April 2023).

### 2.5. Data Visualization

For data visualization, built-in R functions or packages ggplot2 [43], pheatmap [44], and reshape2 [45], were used. Values are represented as mean ± SEM (standard error of the mean). Figure panels were prepared using corelDRAW 2022 v24.1.0360 (Corel Corporation, Ottawa, ON, Canada). Some of the results presented here are part of Karina Barrreiro’s dissertation which is accessible in the Digital Repository of the University of Helsinki (HELDA).

## 3. Results

Our study focused on the kidney-linked and putative reference RNAs in uEV isolates targeting applicability for biomarker discovery. The uEV isolates used to generate the eleven sequencing datasets analyzed in this study were comprehensively characterized in our original publications (Table 1) by electron microscopy, Western blotting, and nanoparticle tracking, RNA fragment length and protein analysis. Briefly, this quality control indicated that the main population of uEV and RNA was small in size and length (<300 nm and <300 nt, respectively) and that the presence of e.g., remnant Tamm-Horsfall protein varied, but was not extensive.

### 3.1. Effect of PreAnalytical Variables on Kidney Transcriptome in uEV Isolates

In previous studies we determined that some preanalytical variables such as storage temperature affect the global uEV transcriptome [13,14]. As the uEV have shown potential as “liquid kidney biopsy” [9], we now assessed whether these preanalytical variables impact the kidney transcriptome in uEV isolates. Here we analyzed the expression level of “kidney-RNAs” i.e., top kidney expressed miRNAs and kidney enriched mRNAs.

#### 3.1.1. Effect of Storage Temperature

**Figure 1:**
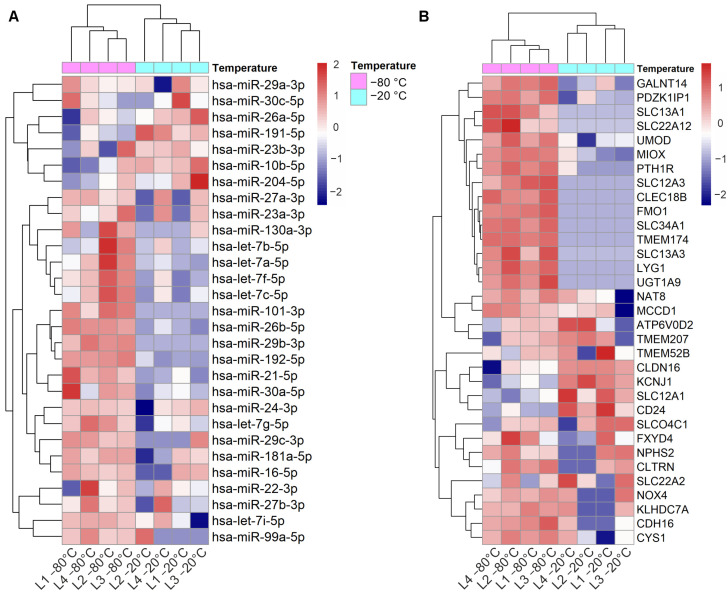
Effect of storage temperature on kidney top expressed miRNAs and kidney enriched genes in uEV isolates. Urine EV were isolated by UC from urine stored at −20 °C vs. −80 °C. (**A**): Heatmap depicts the expression level of 29 out of 40 top miRNAs expressed in kidney (miRNATissueAtlas2) and found in the uEV isolates (miRNAs with ≥1 raw count in at least 50% of the samples). (**B**): Heatmap depicts the expression of 33 out of 53 kidney enriched genes (human protein atlas) found in the uEV isolates (genes with ≥5 raw counts in at least 50% of the samples). Micro RNA (miRNA), messenger RNA (mRNA), ultracentrifugation (UC), urinary extracellular vesicles (uEV).

To analyze the effect of urine storage temperature on miRNAs that have high expression in the kidney, we focused on the top 40 kidney expressed miRNAs. In our dataset (n = 8 samples), we found 29 out of the 40 miRNAs and for 22 of those, the normalized expression level was lower in urines stored at −20 °C than in urines stored at −80 °C (Figure 1). Of note, two of the miRNAs were not detected at all in the −20 °C samples (Table S2).

Out of 56 kidney enriched mRNAs we found 33 in our dataset. Analysis of the expression levels showed that 15 mRNAs were poorly represented in urines stored at −20 °C compared to the ones stored at −80 °C (Figure 1A). Importantly, 10 of the mRNAs were not detected at all in the −20 °C samples (raw counts = 0) (Table S2).

#### 3.1.2. Effect of Isolation Workflows

**Figure 2:**
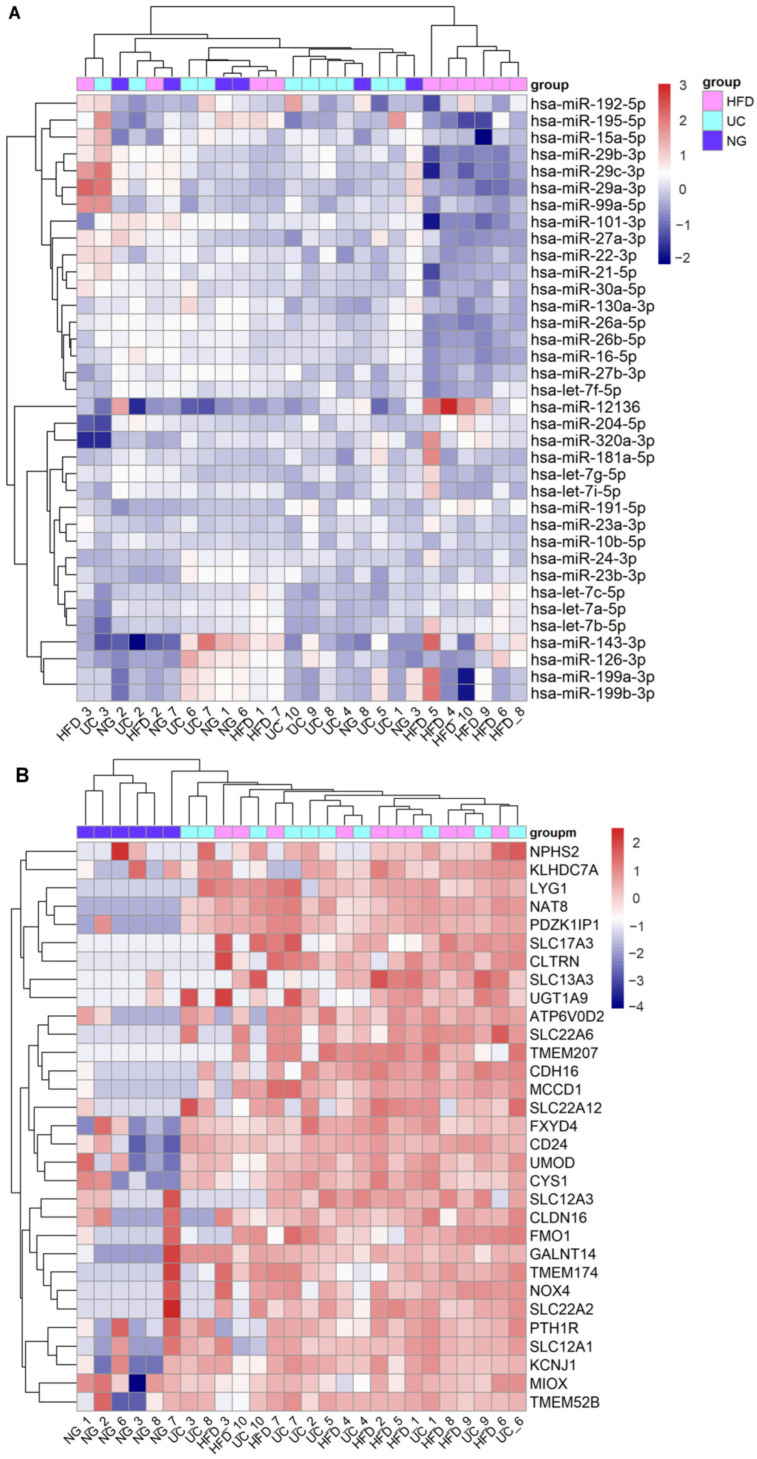
Effect of EV isolation workflows on kidney top expressed miRNAs and kidney enriched genes in uEV isolates. Urine EV were isolated by HFD, NG and UC workflows from urine samples of healthy controls (n = 5) and T1D patients with macroalbuminuria (n = 5). (**A**). Heatmap depicts the expression level of 36 out of 40 top miRNAs expressed in kidney (miRNATissueAtlas2) and found in the uEV isolates (miRNAs with ≥5 raw counts in at least 50% of the samples). (**B**). Heatmap depicts the expression level of 31 out of 56 kidney enriched genes (Human protein atlas) found in the uEV isolates (genes with ≥5 raw counts in at least 50% of the samples).

We next analyzed the effect of the EV isolation workflows on the uEV expression of kidney-RNAs. Out of 40 highly expressed miRNAs of the kidney, we found 36 in our datasets (n = 26 samples). All the miRNAs were stably expressed across the different isolation workflows but the expression of 18 miRNAs was lower in samples from HFD workflow (Figure 2A). We then analyzed differences in the normalized counts of these 18 miRNAs between HFD and UC samples (samples that showed low expression in HFD (4,5,6,8,9,10) and we observed that the normalized counts were systematically lower in HFD samples compared to UC, with differences ranging between 3–58%. MiRNAs with highest differences (>35%) were hsa-miR-101-3p, hsa-miR-26a-5p, hsa-miR-26b-5p, hsa-miR-27a-3p, hsa-miR-29c-3p. Regarding the kidney enriched genes, we found 31 out of the 56 and all of them had lower expression in samples from Norgen urine Exosome Purification and RNA Isolation Midi Kit (NG) (Figure 2B). Five of the mRNA were not detected in any of the NG samples (raw counts = 0) and generally, many of the samples had raw count 0 (Table S3).

Both temperature and isolation workflow impacted the kidney transcriptome in uEV isolates and these differences are in some cases better captured by analyzing kidney-RNAs than by analyzing global expression.

### 3.2. Dysregulated miRNAs in Samples Stored at Suboptimal Temperature: Significance for Kidney Disease Biomarker Discovery

Previously, we reported different miRNA profiles from uEV isolated from urines stored at −20 °C vs. −80 °C [14] (from now on, for simplicity, we will refer to these samples as “urines stored at −20 °C or −80 °C”). Specifically, by differential expression analysis of normalized counts, we found 29 downregulated and 4 upregulated uEV miRNAs in urines stored at −20 °C compared to the ones stored at −80 °C. To assess the biological relevance of the dysregulated miRNAs, we performed a literature review and found that 25/33 miRNAs were associated with kidney diseases (Table 2). In addition, a careful comparison of the raw and normalized counts revealed that most of the downregulated miRNAs in urines stored at −20 °C failed to be detected (raw counts = 0), while the 4 downregulated miRNAs in urines stored at −80 °C were stably expressed across samples and had high raw counts (Table 2 and Table S1). Thus, in urines stored at −20 °C, a significant number of potential kidney disease markers were lost, and the upregulated genes’ raw counts were actually lower than in urines stored at −20 °C. −80 °C samples.

### 3.3. Replication of DKD–Associated miRNA by UC–Based uEV Isolation and Sequencing Workflow

Prior research has reported many miRNAs that associate with DKD in T1D and/or T2D. Thus, we carried out a literature search to generate a list of these DKD -linked miRNAs (padj < 0.05 or *p* < 0.05 and other evidence of association, see methods) and used it for studying their expression in the UC–isolated uEV from DKD patients vs. heathy controls (n = 10 samples). We found (i) 107 miRNAs based on evidence from tissue (human or disease models) or in vitro models and (ii) 63 miRNAs based on evidence from human urine, urinary sediments or uEV (Table 3 and Table 4). MiRNAs dysregulated in tissue or in vitro models were associated to previously described DKD pathways including inflammation, fibrosis, podocyte injury, and oxidative stress (Table 3). We found 12 miRNAs in common between miRNAs deregulated in tissue or in vitro and urine/urine sediments or uEV (highlighted in bold text in Table 4), namely hsa-miR-214-3p, hsa-miR-192, hsa-miR-200c, hsa-miR-15b-5p, hsa-miR-30c-5p, hsa-miR-30b-5p, hsa-miR-21-5p, hsa-miR-30e-5p, hsa-miR-200c-3p, hsa-miR-200a-3p, hsa-miR-155-5p and hsa-miR-29b-3p, which have been shown to modulate hypertrophy, fibrosis, inflammation, and apoptosis.

**Figure 3:**
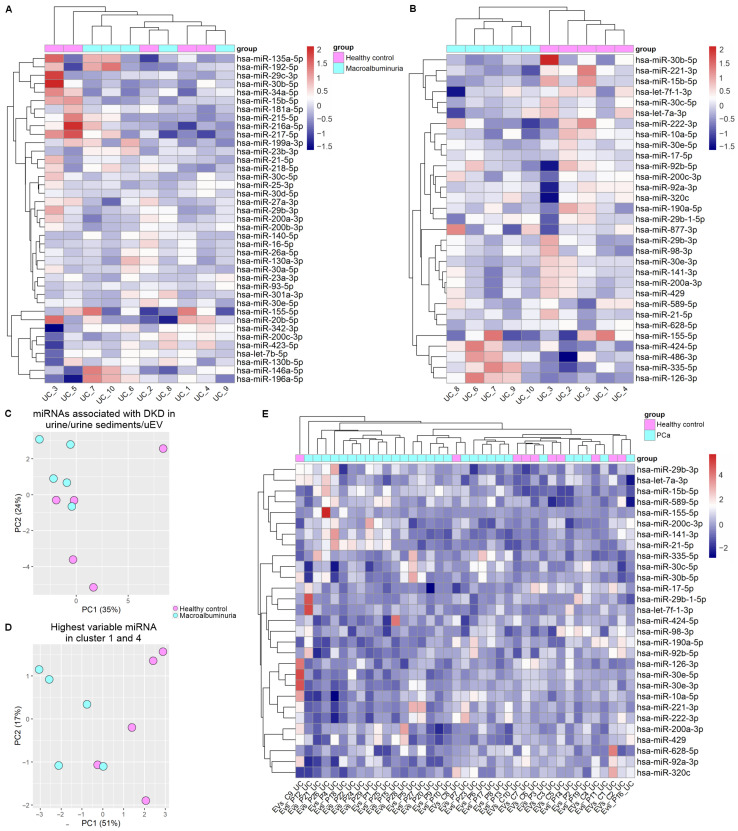
Urinary EV capture miRNAs associated with DKD. (**A**,**B**,**E**). Expression Heatmaps depict the expression of miRNAs associated with DKD and expressed in our uEV datasets. (**C**,**D**). Depict principal component analysis. (**A**). MiRNAs with evidence of dysregulation in kidney tissue and or cell lines and (**B**,**C**). miRNAs dysregulated in uEV/urine/urinary sediments. (**D**). MiRNAs with the highest fold changes from figure B which are part of the first and fourth cluster. The uEV expression data used in (**A**–**D**) corresponds to the UC isolation workflow dataset comprising healthy control and T1D macroalbuminuria groups ([13], part of our UC miRNA dataset in Table 3 and Table 4). (**E**). The 31 miRNAs that could separate individuals with DKD and macroalbuminuria (as shown in B) were analyzed in the PCa uEV miRNA dataset [6]. Diabetic kidney disease (DKD), Prostate cancer (PCa), ultracentrifugation (UC), urinary extracellular vesicles (uEV).

We analysed the expression levels of the miRNA from the literature review (i.e., Table 2 and Table 3) in our uEV data (UC isolation workflow dataset) using expression heatmaps and checked whether the miRNAs could cluster the healthy control and macroalbuminuria groups separately by hierarchical clustering. Our uEV set showed expression of 39 out of 107 miRNAs (36%) dysregulated in DKD with evidence from tissue and in vitro studies, but they did not separate the groups (Figure 3A). However, our uEV set showed expression of a higher proportion of miRNAs—31 out of 63 (49%)—that were dysregulated in DKD with evidence from urine, urine sediment or uEV. Importantly, this set of miRNAs could divide the DKD and healthy control groups into separate clusters and this was observed both by hierarchical clustering and principal component analysis (Figure 3B,C). We focused on the miRNAs with the biggest fold changes that were located on the first and fourth (last) cluster of the heatmap in Figure 3B—they separated the groups by principal component analysis even more evidently than the 31 mRNAs (Figure 3D). From those miRNAs, we compared the direction of change between the literature review and our dataset. For the first cluster, miR-30b-5p, miR-221-3p, let-7f-1-3p, and let-7a-3p followed the same direction of change in both i.e., downregulated in DKD. In contrast, miR-15b-5p was upregulated in the literature with evidence from uEV/urine or urine sediments but downregulated in our uEV dataset. For the fourth cluster, all miRNAs (miR-424-5p, miR-486-3p, miR-335-5p, miR-126-3p) had the same direction of change than what was found in the literature i.e., upregulated in DKD. Moreover, all the miRNAs had evidence of association with DKD in vitro or in vivo and/or association with DKD pathways (in kidney or other cells) (Table 5).

To assess whether these 31 miRNAs would show some specificity for DKD, we carried a similar analysis using our uEV PCa dataset. Supporting DKD specificity, the analysis did not separate the PCa patients from healthy controls (Figure 3E).

Taken together, despite variability between experimental setups, some of the uEV/urine/urine sediment miRNAs presenting candidate markers associated with DKD in the literature were confirmed in our uEV dataset and expression level changes between experimental groups were concordant.

### 3.4. Exploratory Analysis of Reference mRNAs in uEV

**Figure 4:**
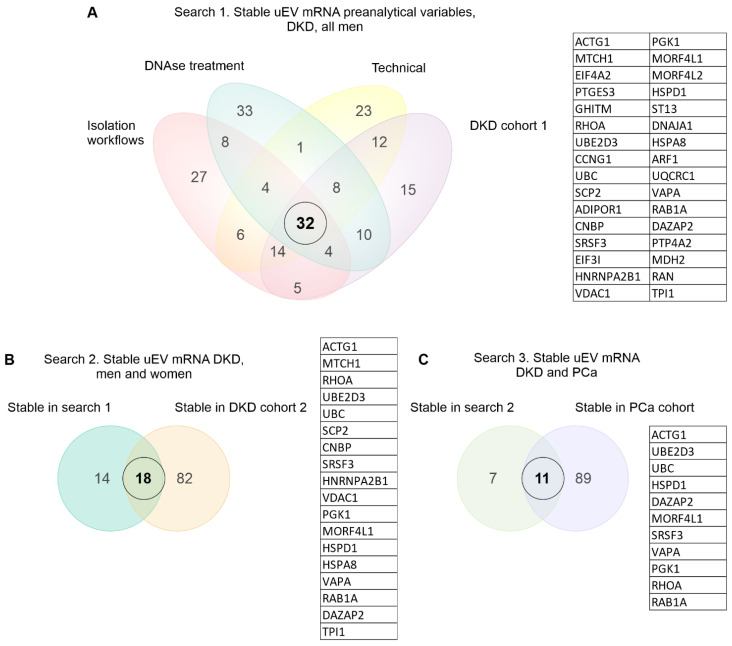
Stable mRNA in common across diverse uEV datasets. Venn diagrams depict elements in common between the different datasets. (**A**–**C**). A 3-step search for the stable uEV mRNA using the top 100 genes with the lowest CV from each dataset. Diabetic kidney disease (DKD), prostate cancer (PCa), urinary extracellular vesicles (uEV).

To select the most stable mRNAs that could serve as candidate reference genes we first focused on uEV samples from our DKD studies that included men only. This choice was due to expected and higher sample-linked [9] and also biological heterogeneity in the women’s cohorts. Datasets were analyzed separately to avoid batch effects i.e., isolation workflows (n = 20 samples), in-column DNAse treatment (n = 19 samples), technical dataset (type of collection, pre-clearing, and replicability, see methods) (n = 39 samples), and DKD cohort 1 (T1D, men) (n = 72 samples). Of note, NG isolation workflow data and storage temperature dataset were excluded from the analysis due to the low expression level of many mRNAs (for raw counts, see Table S3). The top 100 uEV genes with the lowest CV were selected from each dataset and the genes overlapping between all of them were selected for further analysis. We found 32 uEV genes in common between the datasets (Figure 4A).

We next expanded our reference gene analysis to check the stability of expression including women’s uEV samples. Here we searched genes in common between the DKD male (32 uEV stable mRNAs from first search) and DKD cohort 2 (T2D, women) (n = 30 samples) using again the top 100 uEV RNA with low CV (in cohort 2), which showed 18 mRNAs in common (Figure 4B). Finally, we assessed whether some of these 18 mRNAs could also be found from the PCa dataset (n = 8 samples) listing the top 100 uEV mRNAs with low CV. This analysis showed 11 mRNAs in common (HSPD1, SRSF3, VAPA, RAB1A, MORF4L1, PGK1, RHOA, UBE2D3, DAZAP2, UBC, ACTG1) with low CV (Figure 4C, Table 6).

**Figure 5:**
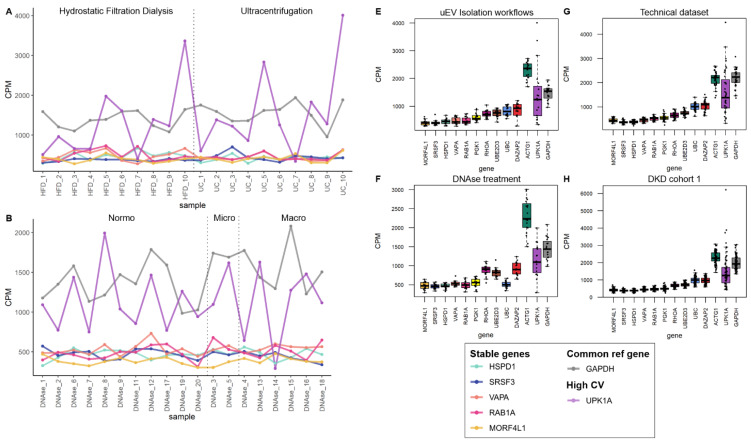

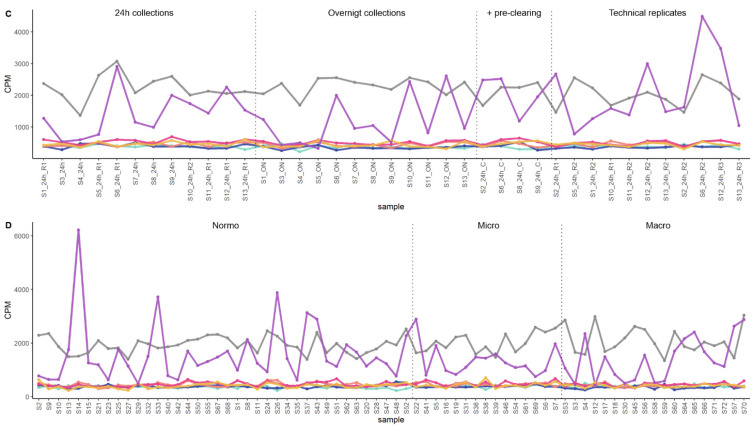
The mRNA sequencing read counts of candidate reference genes in pre-analytical and DKD uEV datasets from men. The uEV datasets included healthy controls and individuals with type 1 diabetes and different stages of albuminuria as well as comparisons of preanalytical variables (all male). (**A**–**D**). Line graphs depicts CPM of HSPD1, SRSF3, VAPA, RAB1A and MORF4L1 across samples and (**E**,**F**). Boxplots depict CPM per candidate reference genes. A reference gene used commonly for normalization (GAPDH) and a gene with high CV in all datasets (UPK1A) were included. (**A**,**E**). EV isolation workflows, (**B**,**F**). In column DNAse treatment during uEV RNA extraction, (**C**,**G**). A technical dataset (type of urine collection, pre-clearing the urine before freezing, and technical replicates) and (**D**,**H**). DKD cohort 1. Sample pairs or triplicates are named similarly apart from the abbreviation of the tested variable. Centrifuged (C), Coefficient of variation (CV), counts per million (CPM), hydrostatic filtration dialysis (HFD), macroalbuminuria (Macro), microalbuminuria (Micro), normoalbuminuria (Normo), ultracentrifugation (UC).

**Figure 6:**
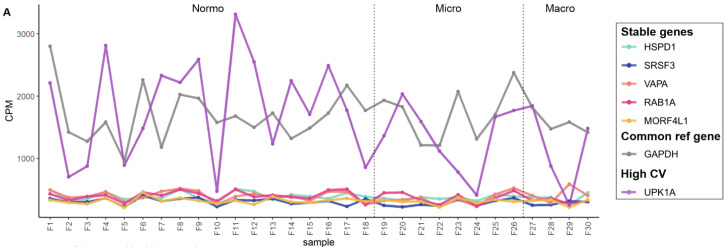

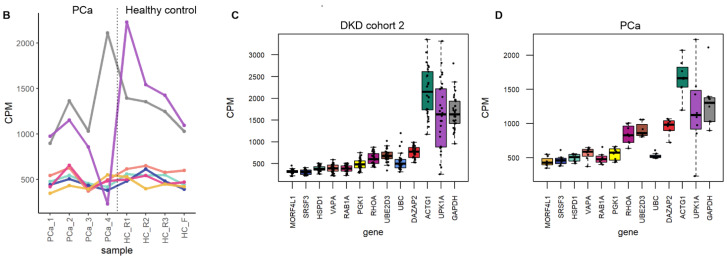
The mRNA sequencing read counts of the candidate reference genes in uEV datasets from DKD study of women and from prostate cancer patients. (**A**,**B**). Line graphs depicts CPM of HSPD1, SRSF3, VAPA, RAB1A and MORF4L1 across samples and (**C**,**D**). Boxplots depict CPM per candidate reference genes. A reference gene used commonly for normalization (GAPDH) and a gene with high CV in all datasets (UPK1A) were included. The uEV datasets included A. DKD cohort 2 (women with type 1 diabetes and different stages of albuminuria) and B. PCa patients and healthy controls (technical replicates, R1-3). Samples PCa1, 3 and 4 were obtained before prostatectomy. Sample PCa2 was obtained after prostactectomy from the same donor as PCa1. Coefficient of variation (CV), counts per million (CPM), macroalbuminuria (Macro), microalbuminuria (Micro), normoalbuminuria (Normo), prostate cancer (PCa).

We analyzed the counts per million (CPM) of the stable genes across samples. In addition, we included GAPDH, a commonly used reference gene, and a gene with high CV (UPK1A) for comparison (Table 6). CPM analysis showed that CPM variation of ACTG1 across samples was similar to the variation observed for GAPDH (both with high and comparable CPM, the rest of the stable mRNA had lower CPM than ACTG1 and GAPDH) but in both cases the variation was low compared to the gene with the highest CV (UPK1A) in all datasets (Figure 5, Figure 6, Figures S1 and S2). For visualization of CPM values across samples, the candidate reference genes were sorted by decreasing standard deviation (SD) value. The 5 genes with the lowest SD value are plotted in Figure 5 and Figure 6A–D and the remaining 6 genes are plotted in Figures S1 and S2A,B. We also summarized the data in boxplots to visualize the CPM dispersion per gene (Figure 5E–H and Figure 6C,D).

**Figure 7:**
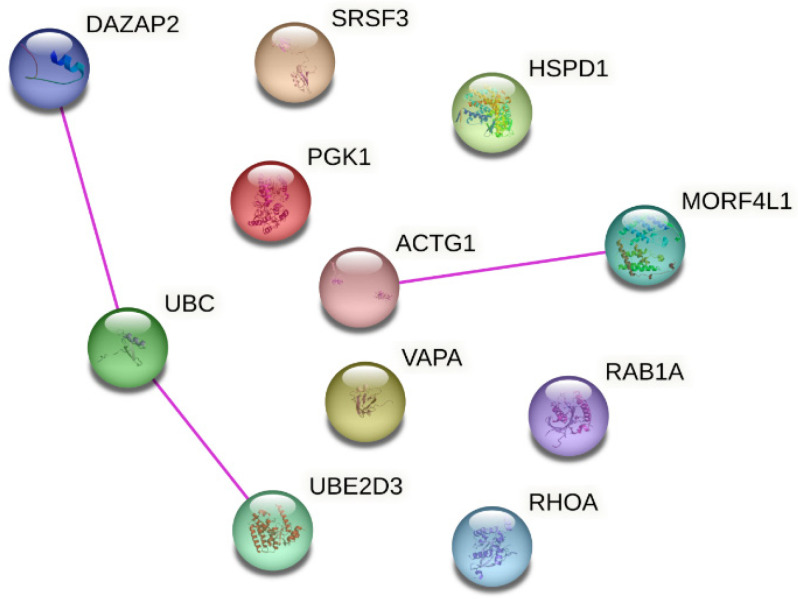
Protein-protein interaction network analysis for the reference gene candidates. Network was generated using STRING (https://string-db.org/, accessed on 28 April 2023) and reproduced under Creative Commons BY 4.0 license (https://creativecommons.org/licenses/by/4.0/, accessed on 28 April 2023). Only interactions with experimental validation evidence in the literature are shown.

It has been suggested that a combination of reference genes could provide a more reliable and accurate normalization approach compared to individual reference genes. For generating such a normalization factor, it is important that genes are not co-regulated. In order to spot genes that may be co-regulated, we examined the functions and associated biological processes of the stable genes. As shown in Table 7, the reference gene’s functions (at the protein level) are varied including protein folding, glycolysis, signaling cascades, intracellular vesicular trafficking, and splicing. They also participate in several different prominent pathways. Of note, UBE and UBE2D3 both ubiquitylate proteins. Moreover, an analysis of protein interaction (based on experimental evidence from literature) using STRING showed interaction of UBC with UBE2D3 and DAZAP2 and of MORF4L1 with ACTG1 (Figure 7). In addition, RHOA is involved in some biological processes shared with other stable genes i.e., with RAB1A (cell migration and substrate adhesion-dependent cell spreading), ACTG1 (response to mechanical stimulus and regulation of focal adhesion assembly), DAZAP (positive regulation of protein serine/threonine kinase activity), and VAPA (positive regulation of I-kappaB kinase/NF-kappaB signaling) (Table 7).

Further we analyzed the stability of the candidate reference genes in datasets from samples that did not perform well in mRNA sequencing i.e., urines stored at −20 °C and uEV isolated with NG isolation workflow. We found that all genes were less stable in samples stored at −20 °C and in NG isolates (Figure S3). Of note, in many NG samples the candidate reference genes were not detected. Despite of this, HSPD1, SRSF3, VAPA, RAB1A, MORF4L1, PGK1, RHOA, UBE2D3, DAZAP2, UBC, ACTG1, showed to be stable in all other diverse experimental conditions and across disease groups. Thus, they may serve as reference genes for uEV mRNA related research.

## 4. Discussion

Urinary EV have been regarded as a promising source of biomarkers [5] and this idea is getting support from an increasing number of studies reporting candidate markers for diseases of diverse etiology [8,9,28,162,163]. However, many obstacles prevent replication of biomarker results and, as a consequence, clinical translation. In this study, we approached three of these obstacles: urine storage, uEV isolation and reference genes in kidney disease transcriptomic research.

The first obstacle is the lack of guidelines to handle and store urine. Urine storage temperature (−20 °C vs. −80 °C) has been shown to affect the size and concentration of uEV [164] and recovery of uEV protein markers but the latter could be sorted out by vortexing samples after thawing [165]. In addition, qPCR-based research has been done on uEV RNA by comparing storage temperatures—including −80 °C, 4 °C, room temperature and 37 °C with variable results [166,167,168]. Our group showed that the global uEV miRNA and mRNA profiles were affected when urines were stored at −20 °C vs. −80 °C [14] and we found sets of downregulated and upregulated genes. As particularly the −20 °C downregulated genes were involved e.g., in carbohydrate or lipid metabolism, the result suggested that −20 °C stored samples are less useful for studying kidney diseases. Here, analyzing further the data, we found that a striking 75% of the −20 °C downregulated miRNAs were associated with various kidney diseases (Table 2). Thus, the result reinforces the idea of avoiding urine samples stored at suboptimal temperatures [14], because such samples might not contain putative valuable disease markers anymore.

We also observed that despite the normalized differential expression, miRNAs that were up-regulated in samples stored at −20 °C had still lower raw counts than the same miRNAs in −80 °C stored samples. Thus, the result was the opposite than what the normalized counts showed. TMM normalization is a method based on library size that uses scaling of raw reads to render library sizes comparable which is needed for differential expression analysis [169]. Considering that the library size of the −20 °C samples was smaller (higher number of 0 raw counts and lower expression in general) than that of the −80 °C samples, the upregulation of miRNAs in −20 °C samples may be an artifact of the data analysis. Further, we showed that kidney-RNAs were detected in small quantities after storage at −20 °C (Figure 1). In particular, kidney enriched mRNAs in uEV isolates were highly affected since almost one third (30%) of them were not detected at all in samples stored at −20 °C. Our results agree with and provide further support to a set of urine storage guidelines that has been published recently [17].

The second obstacle is the lack of standardization of uEV isolation methods. Currently, many isolation principles and workflows are available [170] and it is well known that they typically produce different results [13,15,16,168,171]. Obviously, this represents a problem for study comparisons, even if reporting guidelines now help to identify differences, facilitate replication and/or explain the lack of it [12,172]. Prior studies have explored the effect of uEV isolation workflows on uEV RNA sequencing profiles focusing on miRNA sequencing [15,20,21,22]. We have previously demonstrated that the uEV isolation workflow (UC, HFD and NG) has a surprisingly variable impact on the miRNA and mRNA profiles [13]. Specifically, global miRNA profile analysis suggested that the three workflows were similar overall or—at least—did not differ systematically. This was in contrast to the global mRNA results, where UC and HFD were similar while NG clustered separately [13]. Here, by analyzing the top expressed miRNAs of the kidney, we found that the expression of 18 miRNAs was lower for a set of HFD samples compared to UC and NG samples (Figure 2). While for 13 miRNAs the differences in the expression levels between UC and HFD were small (3–35%) and could be related to technical bias, for 5 miRNAs differences were in the range of 35–55% and could represent real differences. Of note, miRNA hsa-miR-101-3p (a top kidney expressed miRNA) was significantly downregulated in HFD relative to UC samples [13]. The observation that methods capture slightly different kidney enriched miRNAs could be explained, at least partly, by differences in the uEV populations and/or non-EV components captured by the isolation workflows [13]. On the other hand, analysis of kidney enriched mRNAs was consistent with the global analysis i.e., NG samples could not capture these genes as well as UC and HFD (Figure 2). Thus, this shows that for a specific research topic like kidney research, it is best to evaluate the differences between methods using specific end-point targets (kidney-RNAs) in addition to a global level analysis.

In addition to urine storage temperature and uEV isolation workflows, many other preanalytical experimental conditions impact the analytical endpoints as well [12]. As experimental set-ups can differ greatly between studies [18], biomarker results cannot be replicated hindering translation of findings to clinic [173]. Considering all the variability, we were positively surprised that our UC workflow replicated some of the previous results from DKD miRNA studies using uEV or urine/urine sediments (see Table 4) i.e., a set of the miRNAs separated experimental groups (healthy controls vs. T1D with macroalbuminuria) (Figure 3). Further, specifically eight miRNAs followed the same regulation direction in both the literature and our dataset. Three of the eight miRNAs had also evidence of DKD-linked dysregulation in kidney or plasma of individuals with DKD and two showed to be dysregulated in kidney cell lines under hyperglycemic conditions in vitro (in addition to evidence in urine/uEV) and all of them related to pathological mechanisms in DKD such as fibrosis and impaired autophagy [174,175,176] (Table 5). However, our dataset had a low number of samples and thus replication of findings in bigger and more varied cohorts is still needed. Interestingly, we found 12 miRNAs in common between the two literature review generated DKD miRNA lists. These miRNAs were associated with pathological pathways involved in DKD such as hypertrophy and fibrosis. These results suggest that the uEV capture a specific subset of DKD-associated miRNA reflecting the differences in the tissue.

The third obstacle jeopardizing the biomarker replication is the lack of normalizers—in the EV transcriptomics field, this means lack of stable reference genes across e.g., many preanalytical workflows and disease conditions. Urinary EV reference genes are a poorly explored topic. While some recommendations exist on how to select reference genes or normalize gene expression data [29,30,177], there are only few studies on this topic in urine. GAPDH, a commonly used reference gene, and UBC were the most stable in EV derived from liver and breast cancer cell lines [33]. In contrast, Singh et al. (2022) tested five common reference genes (including GAPDH) and found that B2M and RPL13 were the most stable in uEV isolated using PEG from patients with renal graft dysfunction. Thus, the stability of GAPDH appears to be dependent on the disease, biofluid and/or EV isolation method. In this study, using datasets available in our original publications [6,9,13,14], we discovered 11 mRNAs (HSPD1,SRSF3, VAPA, RAB1A, MORF4L1, PGK1, RHOA, UBE2D3, DAZAP2, UBC, ACTG1) that were stable across datasets including different pre-analytical conditions, men and women, healthy controls, T1D and T2D patients with different albuminuria status; and prostate cancer patients (Figure 4, Figure 5, Figure 6, Figures S1 and S2). However, in poor quality sequencing datasets (urine stored at −20 °C and NG isolation workflow), the candidate genes showed poor stability i.e., high CV (Figure S3). Of note, our finding regarding UBC stability in uEV is concordant with findings of Gorgi Bahri (2021) in cell culture media derived EV. Further, even though GAPDH was not one of the most stable mRNAs, it was less variable than UPK1A (a highly variable mRNA selected to compare our candidate reference mRNAs).

One of the reasons that prevents the study of uEV reference genes is the lack of data. Many studies have focused on miRNA/small RNA sequencing, but only few on RNA sequencing. Moreover, the few studies with a good amount of uEV samples from patients [59,178] do not have the associated raw sequencing data and/or raw sequencing counts freely available (to date). Local regulations could hinder the publication of sequencing data but raw count data describing all the pre-processing and alignment procedures is also helpful for the research community. Such practicalities should be considered for the informed consent and ethical permissions. Given more available datasets in future, the stability of our 11 candidate mRNAs could be further tested and a combination of selected genes used as reference genes e.g., by calculating the geometrical mean [179]. As it is recommended that reference genes should belong to different biological pathways and that expression is regulated independently for each reference gene, caution should be taken if using UBC and UBED3 and/or DAZAP2 or MORF4L1 and ACTG1 together since an analysis using STRING showed evidence of experimentally validated interactions in the literature (Figure 7). In addition, UBC and UBE2D3 are co-expressed (https://string-db.org/, accessed on 28 April 2023) and form a protein complex [180]. Moreover, RHOA shared biological processes with RAB1A, ACTG1, DAZAP and VAPA (Table 7). It is good to keep in mind that the uEV reference mRNA candidates could be contributing to biological processes associated with kidney diseases e.g., RAB1A contributes to autophagy. Nevertheless, their stability in our datasets which included isolates from healthy and type 1 diabetic individuals with and without DKD, and diverse preanalytical setups (roughly 200 isolates) motivates further experimental validations.

We acknowledge that a full understanding of the effect of all pre-analytical choices and pathophysiological conditions for transcriptomic applications calls for big testing resources. Ideally, cross-laboratory testing should be performed, and laboratories could implement reference materials, a gold standard isolation protocol, and housekeeping normalizers. Our results here help towards this goal by providing new insights for the three key obstacles hindering uEV biomarker validation. For the first two, urine storage and uEV isolation, we found that it is important to study the raw counts in addition to the normalized counts and kidney-RNAs in addition to the global transcriptome—they offer different although complementary results. For the third, the reference genes, we provide 11 mRNAs that could be tested for qPCR normalization in the context of DKD and prostate cancer. Finally, despite the known and hereby addressed variability between uEV studies, we successfully replicated many previously found urine/uEV/urinary pellet miRNAs associated with DKD in our UC DKD dataset. We regard this as an encouraging result for the reproducibility of uEV biomarker research.

## Figures and Tables

**Table 1:** Datasets included in this study. Diabetic kidney disease (DKD), hydrostatic filtration dialysis (HFD), overnight (ON), prostate cancer (PCa), ultracentrifugation (UC), urinary extracellular vesicles (uEV), Urine Exosome Purification and RNA Isolation Midi Kit (NG). * NG was not included in the analysis due to the poor performance on RNA sequencing. ** 24 h urine collections were not pre-cleared and ON urine collections were pre-cleared.

Study	Description	Storage Temp	PI *	Pre-Clearing	Isolation Method	Urine Sample Type and Disease	n (Donors)	Analysis Type	Reference
Isolation workflows	uEV were isolated from urines	−80 °C	yes	no	UC, HFD, and NG	24 h urine samples from healthy controls and T1D patients with macroalbuminuria. All men.	healthy controls = 5 macroalbuminuria = 5	miRNA and mRNA sequencing	[13]
Storage temperature	uEV were isolated from paired urine aliquots stored at −20 °C or−80 °C.	−80 °C/−20 °C	yes	no	UC	24 h urine samples from T1D patients with normoalbuminuria, microalbuminuria or macroalbuminuria. All men.	normoalbuminuria = 2 macroalbuminuria = 2	miRNA and mRNA sequencing	[14]
DNAse treatment	uEV RNA was isolated adding an in-column DNAse digestion step.	−80 °C	yes	no	UC	24 h urine samples from T1D patients with normoalbuminuria, microalbuminuria or macroalbuminuria. All men.	normoalbuminuria = 11 microalbuminuria = 2 macroalbuminuria = 6	mRNA sequencing	[14]
ON/24 h	uEV were isolated from urines derived from donors that provided on the same day 24 h urine (full void during 24 h) or ON urine (full first void).	−80 °C	yes	no	UC	ON and 24 h urine samples from healthy controls and T1D patients with normoalbuminuria or macroalbuminuria. All men.	ON/24 pairs = 12	mRNA sequencing.	[9]
Pre-clearing	uEV isolated from paired urine aliquots processed +/− pre-clearing before storage.	−80 °C	yes	yes/no	UC	24 h urine samples from T1D patients with microalbuminuria or macroalbuminuria. All men.	pre-clearing pairs = 4	mRNA sequencing	[9]
Replicability of UC workflow	Pairs of urine aliquots were stored and processed at different time points (up to 5 months)	−80 °C	yes	no	UC	24 h urine samples from healthy controls and T1D patients. All men.	Duplicates = 6 Triplicates = 2	mRNA sequencing	[9]
DKD cohort 1	uEV isolated from urines to find candidate markers of DKD.	−80 °C	yes	yes-no **	UC	24 h or ON urine samples from T1D patients with normoalbuminuria, microalbuminuria or macroalbuminuria. All men.	normoalbuminuria = 38 microalbuminuria = 15 macroalbuminuria = 19	mRNA sequencing	[9]
DKD cohort 2	uEV isolated from urines to validate candidate markers.	−80 °C	yes	yes	UC	24 h urine samples from T1D patients with normoalbuminuria, microalbuminuria or macroalbuminuria. All women.	normoalbuminuria = 18 microalbuminuria = 8 macroalbuminuria = 4	mRNA sequencing	unpublished raw count data [9]
PCa cohort	uEV isolated from urine samples from PCa patients	−80 °C	no	yes	UC	Spot urine samples from healthy technical controls and PCa patients before and after radical prostatectomy. Men and a woman.	PCa = 3 healthy controls = 2 (1 man with 3 technical replicates and 1 woman)	mRNA sequencing	[6]

**Table 2:** Down- and up- regulated miRNAs in uEV derived from urines stored at −20 °C for up to 1 year. Acute kidney injury (AKI), chronic kidney disease (CKD), diabetic kidney disease (DKD), lipopolysaccharide (LPS), streptozotocin (STZ), urinary extracellular vesicle (uEV).

		Raw Counts		Normalized Counts (Log2CPM)		
	ID	−80 °C	−20 °C	−80 °C	−20 °C	Association with Kidney Diseases
**Downregulated**	hsa-miR-21-5p	42,760 ± 22,321	230 ± 115	14.5 ± 0.4	12.1 ± 0.4	Dysregulated in DKD in human tissue and DKD models [46,47,48].
	hsa-miR-375	11,496 ± 4880	26 ± 13	12.9 ± 0.1	7.3 ± 2	Pro-apoptotic in an in vitro model of AKI (renal tubular cells) [49].
	hsa-miR-192-5p	10,651 ± 5178	15 ± 8	12.7 ± 0.2	9.5 ± 0.3	Dysregulatedin DKD. Associated with fibrosis [27,50,51].
	hsa-miR-378a-3p	1445 ± 740	10 ± 5	9.9 ± 0.3	3.1 ± 1.6	Upregulation observed in biopsies from donors with glomerular diseases [52].
	hsa-miR-101-3p	971 ± 483	0	8.6 ± 1	1.4 ± 0	Downregulated in kidneys from a mouse diabetic nephropathy model (STZ). Antifibrotic [53].
	hsa-miR-107	700 ± 270	1 ± 0.3	9 ± 0.1	3.7 ± 0.8	Downregulated in kidney biopsies from allograft dysfunction [54].
	hsa-miR-320b	466 ± 262	2 ± 1	8.2 ± 0.3	2.4 ± 1	
	hsa-miR-345-5p	236 ± 121	0	7.2 ± 0.4	1.4 ± 0	Upregulated in urine from a chemical model of AKI in rats [55].
	hsa-miR-328-3p	203 ± 88	0	7.1 ± 0.3	1.4 ± 0	Downregulated in proximal tubule cells that underwent ischemia/reperfusion [56].
	hsa-miR-204-3p	202 ± 157	0	6.3 ± 0.6	1.4 ± 0	Upregulation protected podocytes exposed to high glucose from apoptosis [57].
	hsa-miR-7-5p	174 ± 119	0	6.4 ± 0.7	1.4 ± 0	Downregulation protected proximal tubule cells from LPS in vitro [58].
	hsa-miR-197-3p	154 ± 92	0	6 ± 0.8	1.4 ± 0	Downregulated in urine from donors with intermittent MA [59].
	hsa-miR-20b-5p	151 ± 80	0	6.7 ± 0.6	1.4 ± 0	Downregulated in kidneys and cell lines from mouse models of polycystic kidney disease [60].
	hsa-miR-148a-5p	133 ± 60	0	6.3 ± 0.3	1.4 ± 0	Increased in urine from donors with persistent macroalbuminuria [59].
	hsa-miR-10a-3p	114 ± 79	0	5.9 ± 0.7	1.4 ± 0	Downregulated in kidneys from a mouse model of AKI [61].
	hsa-miR-629-5p	109 ± 81	0	5.5 ± 0.5	1.4 ± 0	Upregulated in kidney biopsies from donors with acute tubular necrosis [62].
	hsa-miR-92a-1-5p	101 ± 59	0	5 ± 0.4	1.4 ± 0	
	hsa-miR-193b-3p	100 ± 62	0	5.8 ± 0.5	1.4 ± 0	Upregulated in kidney from chronic kidney disease biopsies [63].
	hsa-miR-340-5p	99 ± 36	0	6.2 ± 0.3	1.4 ± 0	
	hsa-miR-3065-5p	98 ± 43	0	6.2 ± 0.3	1.4 ± 0	Upregulated in a mouse model of renal fibrosis [64].
	hsa-miR-106a-5p	92 ± 50	0	5.8 ± 0.4	1.4 ± 0	Downregulation associated with podocyte injury induced by high glucose [65].
	hsa-miR-7704	87 ± 37	0	5.8 ± 0.2	1.4 ± 0	
	hsa-miR-324-5p	74 ± 42	0	5.5 ± 0.3	1.4 ± 0	
	hsa-miR-374b-5p	60 ± 24	0	5.5 ± 0.2	1.4 ± 0	Downregulated in diabetic kidney biopsies [66].
	hsa-miR-99b-3p	59 ± 19	0	5.6 ± 0.3	1.4 ± 0	
	hsa-miR-4728-3p	59 ± 20	0	5.5 ± 0.6	1.4 ± 0	
	hsa-miR-132-3p	57 ± 12	0	5.7 ± 0.4	1.4 ± 0	Upregulation increases fibrosis in mouse and in vitro [67].
	hsa-miR-361-5p	55 ± 15	0	5.5 ± 0.6	1.4 ± 0	
	hsa-miR-664a-5p	54 ± 15	0	5.6 ± 0.3	1.4 ± 0	Upregulated in uEV from donors with Idiopathic Membranous Nephropathy [68].
**upregulated**	hsa-miR-10a-5p	47,380 ± 33,187	5272 ± 2636	14.4 ± 0.4	16.7 ± 0.3	Downregulated in urine of individuals with AKI [69].
	hsa-miR-125a-5p	1017 ± 399	103 ± 51	9.3 ± 0.7	12 ± 0.2	Downregulated in urine from donors with membranous nephropathy [70].
	hsa-miR-92b-3p	864 ± 413	58 ± 29	9.1 ± 0.2	11.4 ± 0.3	Upregulated in urine from donors with persistent macroalbuminuria [59].
	hsa-miR-3960	77 ± 44	15 ± 7	4.8 ± 1.1	9.4 ± 0.5	Upregulated in kidney biopsies from donors with acute tubular necrosis [62].

**Table 3:** MiRNAs associated with DKD development and/or progression with evidence in kidney tissue and/or cell lines. Reported target genes for the dysregulated miRNAs have direct regulation evidence (e.g., luciferase reporter assay).

miRNA (Human)	Targeted Genes	Evidence	miRNA Regulation in Disease Group	Dysregulation Effect	Reference
let-7b-5p	Col1a2/4a1	in vitro and in vivo	Down	pro-fibrotic	[71]
miR-15b-5p	BCL-2	in vitro and in vivo	Up	pro-apoptotic	[72]
miR-16-5p	VEGFA	in vitro	Down	pro-apoptotic, podocyte injury	[73]
miR-20b-5p	SIRT7	in vitro	Up	pro-apoptotic	[74]
miR-21-5p	PTEN	in vitro and in vivo	Down	pro-fibrotic (early DKD)	[48]
miR-21-5p	PTEN	in vitro and in vivo	Up	pro-fibrotic	[46]
miR-21-5p	SMAD7	in vitro and in vivo	Up	pro-fibrotic	[47]
miR-21-5p	SMAD7	in vitro and in vivo	-	pro-fibrotic	[75]
miR-21-5p	n.d.	in vitro and in vivo	Up	anti-apoptotic	[76]
miR-21-5p	SMAD7	in vitro and in vivo	Up	pro-fibrotic	[77]
miR-21-5p	Cdc25a, CdK6	in vitro and in vivo	Up	pro-inflammatory, pro-fibrotic	[78]
miR-21	TGF-β, SMAD7, PTEN	in vivo	Up	pro-fibrotic	[79]
miR-22	PTEN	in vitro and in vivo	Up	pro-fibrotic	[80]
miR-23a-3p	SnoN	in vitro	Up	pro-fibrotic	[81]
miR-23b-3p	HMGA2	in vitro and in vivo	Down	pro-fibrotic	[82]
miR-23b	G3BP2	in vitro and in vivo	Down	pro-fibrotic	[83]
miR-25-3p	NOX4	in vitro and in vivo	Down	oxidative stress	[84]
miR-25-3p	NOX4	in vitro and in vivo	Down		[85]
miR-25-3p	PTEN	in vitro and in vivo	Down	pro-apoptotic, increase ROS	[86]
miR-25	CDC42	in vitro and in vivo	Down	pro-fibrotic	[87]
miR-26a-5p	CTGF	in vitro and in vivo	Down	pro-fibrotic	[88]
miR-27a-3p	PPARγ	in vitro and in vivo	Up	pro-fibrotic	[89]
miR-27a	PPARγ	in vitro and in vivo	Up	podocyte injury	[90]
miR-29b-3p	n.d.	in vitro and in vivo	Up		[91]
miR-29b-3p	TGF-β, SMAD3	in vitro and in vivo	Down	pro-fibrotic, pro-inflammatory	[92]
miR-29c-3p	Spry1	in vitro and in vivo	Up	pro-apoptotic, pro-fibrotic	[93]
miR-29a	HDAC	in vitro and in vivo	Down	pro-apoptotic	[94]
miR-29a	n.d.	in vitro and in vivo	Down	pro-fibrotic	[95]
miR-29a/b/c family	Col1a2/4a1	in vitro and in vivo	Down	pro-fibrotic	[96]
miR-30e-5p	GLIPR-2	in vitro and in vivo	Down	pro-fibrotic	[97]
miR-30s (family)	Mtdh	in vitro and in vivo	Down	pro-apoptotic	[98]
miR-30b-5p	SNAI1	in vitro	Down	increased markers of ephitelial to mesenchimal transition.	[99]
miR-30c-5p	ROCK2	in vitro and in vivo	Down	pro-apoptotic, reduced cell proliferation, increased epithelial-mesenchymal transition	[100]
miR-34a-5p	GAS1	in vitro and in vivo	Up	regulated mesangial proliferation and glomerular hypertrophy	[101]
miR-34a-5p	SIRT1	in vitro and in vivo	Up	pro-fibrotic	[102]
miR-34c-5p	Notch1 and Jagged1	in vitro	Down	pro-apoptotic	[103]
miR-93-5p	VEGFA	in vitro and in vivo	Down	angiogenic, pro-fibrotic	[104]
miR-124-5p	n.d.	in vivo	Up	podocyte loss	[105]
hsa-miR-126-3p	n.d.	in vitro and in vivo	Up		[91]
miR-130a-3p	TNF-α	in vitro	Down	oxidative stress, pro-apoptotic	[106]
miR-130b-5p	snail	in vitro and in vivo	Down	pro-fibrotic	[107]
miR-133b	SIRT1	in vitro and in vivo	Up	pro-fibrotic	[108]
miR-134-5p	BCL2	in vitro and in vivo	Up	pro-apoptotic	[109]
miR-135a-5p	TRPC1	in vitro and in vivo	Up	pro-fibrotic	[110]
miR-140-5p	TLR4	in vitro and in vivo	Down	pro-apoptotic, pro-inflammatory	[111]
miR-145-5p	n.d.	in vitro and in vivo	Up		[112]
miR-145-5p	Notch1	in vitro	Down	pro-apoptotic	[113]
miR-146a-5p	n.d.	in vitro and in vivo	Up	pro-inflammatory	[114]
miR-146a-5p	ErbB4, Notch1	in vitro and in vivo	Down	diabetic glomerulopathy and podocyte injury.	[115]
miR-146a	n.d.	in vivo	Down	pro-inflammatory	[116]
miR-146a	n.d.	in vitro and in vivo	Down	oxidative stress	[117]
miR-155-5p	n.d.	in vitro and in vivo	Up	pro-inflammatory	[114]
miR-155-5p	n.d.	in vitro and in vivo	Up		[91]
miR-155-5p	Sirt1	in vitro	Up	reduced autophagy, anti-fibrotic	[118]
miR-181a-5p	Egr1	in vitro and in vivo	Down	pro-fibrotic	[119]
miR-192-5p	Zeb2	in vitro and in vivo	Up	pro-fibrotic	[120]
miR-192-5p	Zeb2	in vitro and in vivo	Down	pro-fibrotic	[51]
miR-192-5p	Zeb2	in vitro and in vivo	Up	pro-fibrotic	[121]
miR-192	n.d.	in vitro and in vivo	Up in microalbuminuria, Down in macroalbuminuria		[27]
miR-192	Zeb1/2	in vitro and in vivo	Down	pro-fibrotic	[50]
miR-192	Zeb2	in vitro and in vivo	Up	pro-fibrotic	[122]
miR-193a	APOL1	in vitro	Up	Podocyte dedifferentiation	[123]
miR-195	n.d.	in vitro and in vivo	Down	anti-apoptotic	[124]
miR-196a-5p	p27(kip1)	in vitro and in vivo	Down	hypertrophy	[125]
miR-199a-3p	IKKβ	in vitro and in vivo	Down	pro-apoptotic, pro-inflammatory	[126]
miR-199b-5p	SIRT1	in vitro and in vivo	Up	pro-fibrotic	[108]
miR-200a-3p	TGF-β2	in vitro and in vivo	Down	pro-fibrotic	[127]
miR-200 b/c-3p	Zeb1	in vitro and in vivo	Up	pro-fibrotic	[121]
miR-200 b/c	FOG2	in vitro and in vivo	Up	Hypertrophy	[128]
miR-214-3p	PTEN	in vitro and in vivo	Up	Hypertrophy	[129]
miR-214-3p	PTEN	in vitro and in vivo	Up	Hypertrophy	[130]
miR-215-5p	Zeb2	in vitro and in vivo	Down	pro-fibrotic	[51]
miR-216a-5p	PTEN	in vitro and in vivo	Up	Hypertrophy, survival	[131]
miR-216a-5p	Ybx1	in vivo	UP	pro-fibrotic	[132]
miR-217-5p	PTEN	in vitro	Up	Defective autophagy, proapoptotic	[133]
miR-217-5p	PTEN	in vitro and in vivo	Up	Hypertrophy, survival	[131]
miR-218-5p	HO-1	in vitro	Up	pro-apoptotic	[134]
miR-301a-3p	TNF-α	in vitro	Down	oxidative stress, pro-apoptotic	[106]
miR-342-3p	SOX6	in vitro and in vivo	Down	pro-fibrotic	[135]
miR-374a	MCP-1	in vitro and in vivo	Down	pro-inflammatory	[136]
miR-377-3p	SOD1/2, PAK1	in vitro and in vivo	Up	pro-fibrotic	[137]
miR-379-5p	LIN28B	in vitro and in vivo	Down	fibrotic	[138]
miR-379 megacluster	EDEM3, ATF3, TNRC6B, CPEB4, PHF21A	in vitro and in vivo	Up	pro-fibrotic	[139]
miR-423-5p	NOX4+D98	in vitro	Down	pro-apoptotic, pro-fibrotic, pro-inflammatory, oxidative stress	[140]
miR-451a	LMP7	in vitro and in vivo	Down	pro-inflammatory	[141]
miR-451a	n.d.	in vivo	Up/Down	anti-fibrotic?	[142]
miR-503	E2F3	in vitro and in vivo	Up	podocyte injury	[143]
miR-770-5p	TIMP3	in vitro and in vivo	Up	pro-apoptotic, pro-inflammatory	[144]
miR-874	LPP3	in vitro and in vivo	Up (overt nephropathy)	pro-fibrotic, anti-apoptotic	[145]
miR-874	TLR5	in vitro and in vivo	Down	pro-inflammatory	[146]
miR-1207-5p	n.d.	in vitro	Up	pro-fibrotic	[147]

**Table 4:** MiRNAs associated with DKD development and/or progression with evidence from urine, urinary sediments or uEV. Chronic kidney disease (CKD), diabetic kidney disease (DKD), intermittent microalbuminuria (IMA), persistent microalbuminuria (PMA), microalbuminuria (MA), type 1 diabetes (T1D), type 2 diabetes (T2D). * Validated with an independent cohort, ** validated with another detection method, in kidney biopsies, in vitro or in a model organism. MiRNAs in common with Table 3 are highlighted in bold text.

Sample	Groups	Upregulated miRNAs	Downregulated miRNAs	Reference
Urine	Urine from T1D (Normal, overt nephropathy, intermittent microalbuminuria, persistent microalbuminuria)	DKD vs. non DKD: miR-619, miR-486-3p, miR-335-5p, miR-552, miR-1912, miR-1124-3p, miR-424-5p, miR-141-3p, miR-29b-1-5p	DKD vs non-DKD: miR-221-3p	[148]
		MA vs. baseline: **miR-214-3p**, miR-92b-5p, miR-765, miR-429, miR-373-5p, miR-1913, miR-638	MA vs. baseline: miR-323b-5p, miR-221-3p, miR-524-5p, miR-188-3p	
		PMA vs. IMA: miR323b-5p, miR-433, miR-17-5p, miR-222-3p, 628-5p	PMA vs. IMA: miR-589-5p, miR-373-5p, miR92a-3p	
Urinary sediments	Diabetic glomerulosclerosis, minimal change nephropathy or focal glomerulosclerosis, membranous nephropathy, and healthy donors	**miR-200c**	miR-638, **miR-192**	[149]
uEV **	T1D with normoalbuminuria and microalbuminuria and non-diabetic controls	miR-130a, miR-145	miR-155, miR-424	[112]
Urine	T2D DKD, T2D, and healthy donors	miR-126 (T2D DKD > T2D)		[150]
uEV	T2D normoalbuminuric, microalbuminuric, or macroalbuminuric	microalbuminuria vs. normoalbuminuria and controls: **miR-192**, miR-194, and miR-215.	macroalbuminuria vs. microalbuminuria: **miR-192**, miR-215	[27]
uEV *	T2D DKD, T2D, and healthy donors	miR-320c, miR-6068		[151]
urine pellets and uEV *	T2D albuminuric, normoalbuminuric, and healthy controls	miR-15b, miR-34a, miR-636		[152]
uEV *	T2D normoalbuminuria and microalbuminuria	miR-877-3p		[153]
uEV **	T1D normoalbuminuria, intermittent macroalbuminuria, persistent macroalbuminuria, and overt macroalbuminuria	Overt vs. normal: miR-26a-1-5p, miR-30-5p PMA vs. IMA/non microalbuminuria: **miR-200c-3p**	Overt vs. normal: miR144-3p	[59]
Urine **		PMA vs. IMA: miR10a-5p, **miR-200a-3p**		
Urine *	Diabetic, DKD and healthy donors	miR-126-3p, **miR-155-5p**, and **miR-29b-3p**		[91]
Urine *	Urine and plasma from T1D and DKD		**miR-30e-5p**	[154]
uEV *	T2D DKD, T2D normal renal function, and non-T2D CKD	**miR-21-5p**	**miR-30b-5p**	[28]
Urine **	DKD and non-diabetic renal disease		T2D vs. non-diabetic renal disease: miR-27-3p, miR-1228	[155]
uEV *	T2D and normoalbuminuria, microalbuminuria or macroalbuminuria and healthy donors	**miR-15b-5p**		[72]
uEV *	TD2 DKD and healthy donors		**miR-30e-3p**, **miR-30c-5p**, miR-190a-5p, miR-98-3p, let-7a-3p, **miR-30b-5p**, and let-7f-1-3p	[156]

**Table 5:** miRNAs dysregulated in uEV/urine/urinary sediments from individuals with DKD. Cluster 1 and 4 (see Figure 3B) miRNAs and association with diabetic kidney disease or diabetic kidney disease associated mechanisms in kidney and/or other cells. Acute kidney injury (AKI), diabetic kidney disease (DKD), type 2 diabetes (T2D), urinary extracellular vesicle (uEV).

		Regulation in UC Dataset	Regulation in uEV/Urine/Urine Sediments Literature	Examples of Association with Diabetic Kidney Disease or Kidney Diseases, or Pathways Associated with DKD (e.g., Fibrosis, Inflammation, Autophagy, and Oxidative Stress)
**Cluster 1**	miR-30b-5p	down	down	In hyperglycemic conditions, expression levels reduced in HK-2 cells and epithelial-mesenchymal transition was increased [99].
	miR-221-3p	down	down	In HUVEC cells, hyperglycemia induced this miRNA and was associated with impairment of endothelial cell migration and homing [157].
	miR-15b-5p	down	up	Upregulated in urine from db/db mouse and T2D patients. In mesangial cell lines hyperglycemia upregulates this miRNA and targets BCL-2 inducing apoptosis [72].
	let-7f-1-3p	down	down	Downregulated in plasma extracellular vesicles from patients with DKD [156].
	let-7a-3p	down	down	Downregulation after exposure to hypoxia in HT-29 cells [158].
**Cluster 4**	miR-424-5p	up	up	Upregulated in high fat diet fed mice and in hepatocytes treated with palmitate. MiR-424-5p suppressed insulin receptor expression in hepatocytes i.e., suggesting a role in insulin resistance [159].
	miR-486-3p	up	up	Downregulated in biopsies from patients with diabetic nephropathy [160].
	miR-335-5p	up	up	In mesangial cells, overexpression of miR-335 induces senescence and increses reactive oxigen species by taregting SOD2 [161].
	miR-126-3p	up	up	Increased in kidney biopsies from patients with DKD [91].

**Table 6:** Coefficient of variation (CV) of the stable genes across datasets. Diabetic kidney disease (DKD), prostate cancer (PCa), type 1 diabetes (T1D). * Commonly used reference gene for normalization of qPCR data. ** Gene with high CV in all datasets.

	CV					
	Isolation Workflows	DNAse Treatment	Technical Datasets	DKD Cohort 1 (T1D, Men)	DKD Cohort 2 (T1D, Women)	PCa
**HSPD1**	0.23	0.13	0.15	0.14	0.16	0.12
**SRSF3**	0.21	0.13	0.17	0.15	0.18	0.16
**VAPA**	0.26	0.13	0.16	0.16	0.23	0.16
**RAB1A**	0.26	0.19	0.15	0.18	0.21	0.17
**MORF4L1**	0.22	0.13	0.17	0.21	0.16	0.16
**PGK1**	0.22	0.20	0.21	0.19	0.24	0.16
**RHOA**	0.17	0.16	0.19	0.15	0.22	0.16
**UBE2D3**	0.18	0.14	0.13	0.15	0.20	0.11
**DAZAP2**	0.26	0.19	0.19	0.20	0.17	0.16
**UBC**	0.19	0.16	0.16	0.20	0.38	0.11
**ACTG1**	0.13	0.19	0.14	0.15	0.25	0.08
GAPDH *	0.18	0.20	0.17	0.19	0.24	0.29
UPK1A **	0.70	0.36	0.59	0.63	0.49	0.49

**Table 7:** Functions and gene ontology biological processes associated with the stable genes (GeneCards, www.genecards.org, accessed on 27 June 2023) and (Uniprot, https://www.uniprot.org/, accessed on 28 April 2023).

Gene Name	Entry	Protein Names	Function	Gene Ontology (Biological Process)
PGK1	P00558	Phosphoglycerate kinase 1	It catalyses the glycolytic pathway conversion of 1,3-diphosphoglycerate to 3-phosphoglycerate. It may act as a co-factor of polymerase alpha.	canonical glycolysis [GO:0061621]; cellular response to hypoxia [GO:0071456]; epithelial cell differentiation [GO:0030855]; gluconeogenesis [GO:0006094]; glycolytic process [GO:0006096]; negative regulation of angiogenesis [GO:0016525]; phosphorylation [GO:0016310]; plasminogen activation [GO:0031639]
UBC	P0CG48	Polyubiquitin-C	Polyubiquitin precursor. Ubiquitination has been associated with processes such as protein degradation, DNA repair, and cell cycle regulation.	modification-dependent protein catabolic process [GO:0019941]; protein ubiquitination [GO:0016567]
HSPD1	P10809	60 kDa heat shock protein, mitochondrial	Member of the chaperonin family. Essential role in folding and assembly of newly imported proteins in the mitochondria.	‘de novo’ protein folding [GO:0006458]; activation of cysteine-type endopeptidase activity involved in apoptotic process [GO:0006919]; apoptotic mitochondrial changes [GO:0008637]; B cell activation [GO:0042113]; B cell proliferation [GO:0042100]; biological process involved in interaction with symbiont [GO:0051702]; cellular response to interleukin-7 [GO:0098761]; chaperone-mediated protein complex assembly [GO:0051131]; isotype switching to IgG isotypes [GO:0048291]; mitochondrial unfolded protein response [GO:0034514]; MyD88-dependent toll-like receptor signaling pathway [GO:0002755]; negative regulation of apoptotic process [GO:0043066]; positive regulation of apoptotic process [GO:0043065]; positive regulation of interferon-alpha production [GO:0032727]; positive regulation of interleukin-10 production [GO:0032733]; positive regulation of interleukin-12 production [GO:0032735]; positive regulation of interleukin-6 production [GO:0032755]; positive regulation of macrophage activation [GO:0043032]; positive regulation of T cell activation [GO:0050870]; positive regulation of T cell mediated immune response to tumor cell [GO:0002842]; positive regulation of type II interferon production [GO:0032729]; protein folding [GO:0006457]; protein import into mitochondrial intermembrane space [GO:0045041]; protein maturation [GO:0051604]; protein refolding [GO:0042026]; protein stabilization [GO:0050821]; response to cold [GO:0009409]; response to unfolded protein [GO:0006986]; T cell activation [GO:0042110]
UBE2D3	P61077	Ubiquitin-conjugating enzyme E2 D3	Member of the E2 ubiquitin-conjugating enzyme family. This enzyme participates of the ubiquitination of proteins.	apoptotic process [GO:0006915]; DNA repair [GO:0006281]; negative regulation of BMP signaling pathway [GO:0030514]; negative regulation of transcription by RNA polymerase II [GO:0000122]; positive regulation of protein targeting to mitochondrion [GO:1903955]; proteasome-mediated ubiquitin-dependent protein catabolic process [GO:0043161]; protein autoubiquitination [GO:0051865]; protein K11-linked ubiquitination [GO:0070979]; protein K48-linked ubiquitination [GO:0070936]; protein modification process [GO:0036211]; protein monoubiquitination [GO:0006513]; protein polyubiquitination [GO:0000209]; protein ubiquitination [GO:0016567]
RHOA	P61586	Transforming protein RhoA	Member of the Rho family of small GTPases. These proteins function as molecular switches in signal transduction cascades.	actin cytoskeleton organization [GO:0030036]; actin cytoskeleton reorganization [GO:0031532]; actin filament organization [GO:0007015]; alpha-beta T cell lineage commitment [GO:0002363]; androgen receptor signaling pathway [GO:0030521]; angiotensin-mediated vasoconstriction involved in regulation of systemic arterial blood pressure [GO:0001998]; aortic valve formation [GO:0003189]; apical junction assembly [GO:0043297]; apolipoprotein A-I-mediated signaling pathway [GO:0038027]; beta selection [GO:0043366]; cell junction assembly [GO:0034329]; cell migration [GO:0016477]; cell-matrix adhesion [GO:0007160]; cellular response to chemokine [GO:1990869]; cellular response to cytokine stimulus [GO:0071345]; cellular response to lipopolysaccharide [GO:0071222]; cerebral cortex cell migration [GO:0021795]; cleavage furrow formation [GO:0036089]; cortical cytoskeleton organization [GO:0030865]; cytoplasmic microtubule organization [GO:0031122]; endothelial cell migration [GO:0043542]; endothelial tube lumen extension [GO:0097498]; establishment of epithelial cell apical/basal polarity [GO:0045198]; establishment or maintenance of cell polarity [GO:0007163]; forebrain radial glial cell differentiation [GO:0021861]; GTP metabolic process [GO:0046039]; kidney development [GO:0001822]; mitotic cleavage furrow formation [GO:1903673]; mitotic spindle assembly [GO:0090307]; motor neuron apoptotic process [GO:0097049]; negative chemotaxis [GO:0050919]; negative regulation of cell migration involved in sprouting angiogenesis [GO:0090051]; negative regulation of cell size [GO:0045792]; negative regulation of cell-substrate adhesion [GO:0010812]; negative regulation of I-kappaB kinase/NF-kappaB signaling [GO:0043124]; negative regulation of intracellular steroid hormone receptor signaling pathway [GO:0033144]; negative regulation of motor neuron apoptotic process [GO:2000672]; negative regulation of neuron differentiation [GO:0045665]; negative regulation of neuron projection development [GO:0010977]; negative regulation of oxidative phosphorylation [GO:0090324]; negative regulation of reactive oxygen species biosynthetic process [GO:1903427]; negative regulation of vascular associated smooth muscle cell migration [GO:1904753]; negative regulation of vascular associated smooth muscle cell proliferation [GO:1904706]; neuron migration [GO:0001764]; neuron projection morphogenesis [GO:0048812]; odontogenesis [GO:0042476]; ossification involved in bone maturation [GO:0043931]; positive regulation of actin filament polymerization [GO:0030838]; positive regulation of alpha-beta T cell differentiation [GO:0046638]; positive regulation of cell growth [GO:0030307]; positive regulation of cysteine-type endopeptidase activity involved in apoptotic process [GO:0043280]; positive regulation of cytokinesis [GO:0032467]; positive regulation of I-kappaB kinase/NF-kappaB signaling [GO:0043123]; positive regulation of leukocyte adhesion to vascular endothelial cell [GO:1904996]; positive regulation of lipase activity [GO:0060193]; positive regulation of neuron apoptotic process [GO:0043525]; positive regulation of neuron differentiation [GO:0045666]; positive regulation of NIK/NF-kappaB signaling [GO:1901224]; positive regulation of podosome assembly [GO:0071803]; positive regulation of protein serine/threonine kinase activity [GO:0071902]; positive regulation of stress fiber assembly [GO:0051496]; positive regulation of T cell migration [GO:2000406]; positive regulation of translation [GO:0045727]; positive regulation of vascular associated smooth muscle contraction [GO:1904695]; regulation of actin cytoskeleton organization [GO:0032956]; regulation of calcium ion transport [GO:0051924]; regulation of cell migration [GO:0030334]; regulation of cell shape [GO:0008360]; regulation of dendrite development [GO:0050773]; regulation of focal adhesion assembly [GO:0051893]; regulation of microtubule cytoskeleton organization [GO:0070507]; regulation of modification of postsynaptic actin cytoskeleton [GO:1905274]; regulation of modification of postsynaptic structure [GO:0099159]; regulation of neural precursor cell proliferation [GO:2000177]; regulation of osteoblast proliferation [GO:0033688]; regulation of systemic arterial blood pressure by endothelin [GO:0003100]; regulation of transcription by RNA polymerase II [GO:0006357]; response to amino acid [GO:0043200]; response to ethanol [GO:0045471]; response to glucocorticoid [GO:0051384]; response to glucose [GO:0009749]; response to hypoxia [GO:0001666]; response to mechanical stimulus [GO:0009612]; response to xenobiotic stimulus [GO:0009410]; Rho protein signal transduction [GO:0007266]; Roundabout signaling pathway [GO:0035385]; skeletal muscle satellite cell migration [GO:1902766]; skeletal muscle tissue development [GO:0007519]; stress fiber assembly [GO:0043149]; stress-activated protein kinase signaling cascade [GO:0031098]; substantia nigra development [GO:0021762]; substrate adhesion-dependent cell spreading [GO:0034446]; trabecula morphogenesis [GO:0061383]; Wnt signaling pathway, planar cell polarity pathway [GO:0060071]; wound healing, spreading of cells [GO:0044319]
RAB1A	P62820	Ras-related protein Rab-1A	Member of the Ras superfamily of GTPases. These proteins act as regulators of intracellular membrane trafficking.	autophagosome assembly [GO:0000045]; autophagy [GO:0006914]; cell migration [GO:0016477]; COPII-coated vesicle cargo loading [GO:0090110]; defense response to bacterium [GO:0042742]; endocytosis [GO:0006897]; endoplasmic reticulum to Golgi vesicle-mediated transport [GO:0006888]; Golgi organization [GO:0007030]; growth hormone secretion [GO:0030252]; intracellular protein transport [GO:0006886]; melanosome transport [GO:0032402]; positive regulation of glycoprotein metabolic process [GO:1903020]; positive regulation of interleukin-8 production [GO:0032757]; substrate adhesion-dependent cell spreading [GO:0034446]; vesicle transport along microtubule [GO:0047496]; vesicle-mediated transport [GO:0016192]; virion assembly [GO:0019068]
ACTG1	P63261	Actin, cytoplasmic 2	Cytoplasmic actin expressed in all cell types.	angiogenesis [GO:0001525]; cellular response to type II interferon [GO:0071346]; maintenance of blood-brain barrier [GO:0035633]; morphogenesis of a polarized epithelium [GO:0001738]; platelet aggregation [GO:0070527]; positive regulation of cell migration [GO:0030335]; positive regulation of gene expression [GO:0010628]; positive regulation of wound healing [GO:0090303]; protein localization to bicellular tight junction [GO:1902396]; regulation of focal adhesion assembly [GO:0051893]; regulation of stress fiber assembly [GO:0051492]; regulation of synaptic vesicle endocytosis [GO:1900242]; regulation of transepithelial transport [GO:0150111]; response to calcium ion [GO:0051592]; response to mechanical stimulus [GO:0009612]; retina homeostasis [GO:0001895]; sarcomere organization [GO:0045214]; tight junction assembly [GO:0120192]
SRSF3	P84103	Serine/arginine-rich splicing factor 3 (Pre-mRNA-splicing factor SRP20)	Member of the serine/arginine (SR)-rich family of pre-mRNA splicing factors. This protein is part of the spliceosome.	cellular response to leukemia inhibitory factor [GO:1990830]; mRNA export from nucleus [GO:0006406]; mRNA splicing, via spliceosome [GO:0000398]; primary miRNA processing [GO:0031053]; regulation of mRNA splicing, via spliceosome [GO:0048024]
DAZAP2	Q15038	DAZ-associated protein 2 (Deleted in azoospermia-associated protein 2)	Proline rich protein that is involved in various biological processes by interacting with proteins such as DAZ and function.	positive regulation of protein serine/threonine kinase activity [GO:0071902]; positive regulation of RNA polymerase II regulatory region sequence-specific DNA binding [GO:1905636]; protein destabilization [GO:0031648]; stress granule assembly [GO:0034063]
VAPA	Q9P0L0	Vesicle-associated membrane protein-associated protein A (VAMP-A)	Transmembrane protein which may involve function in vesicle trafficking, membrane fusion, protein complex assembly and cell motility.	cell death [GO:0008219]; ceramide transport [GO:0035627]; COPII-coated vesicle budding [GO:0090114]; endoplasmic reticulum to Golgi vesicle-mediated transport [GO:0006888]; membrane fusion [GO:0061025]; negative regulation by host of viral genome replication [GO:0044828]; neuron projection development [GO:0031175]; phospholipid transport [GO:0015914]; positive regulation by host of viral genome replication [GO:0044829]; positive regulation of I-kappaB kinase/NF-kappaB signaling [GO:0043123]; protein localization to endoplasmic reticulum [GO:0070972]; sphingomyelin biosynthetic process [GO:0006686]; sterol transport [GO:0015918]; viral release from host cell [GO:0019076]
MORF4L1	Q9UBU8	Mortality factor 4-like protein 1 (MORF-related gene 15 protein)	Involved in transcriptional activation by being part of the NuA4 histone acetyltransferase (HAT) complex.	chromatin organization [GO:0006325]; double-strand break repair via homologous recombination [GO:0000724]; fibroblast proliferation [GO:0048144]; histone acetylation [GO:0016573]; histone deacetylation [GO:0016575]; histone H2A acetylation [GO:0043968]; histone H4 acetylation [GO:0043967]; positive regulation of DNA-templated transcription [GO:0045893]; positive regulation of double-strand break repair via homologous recombination [GO:1905168]; regulation of apoptotic process [GO:0042981]; regulation of cell cycle [GO:0051726]; regulation of double-strand break repair [GO:2000779]

## Data Availability

Datasets used in this publication are available in the original publications mentioned in Table 1.

## Supplementary Materials

The following supporting information can be downloaded at: https://www.mdpi.com/article/10.3390/genes14071415/s1, Figure S1: The mRNA sequencing read counts of six of the eleven candidate reference genes across samples in pre-analytical and DKD uEV datasets from men. Figure S2: The mRNA sequencing read counts of six of the eleven candidate reference genes across samples in uEV datasets from DKD study of women and from prostate cancer patients. Figure S3: The mRNA sequencing read counts of candidate reference genes in storage temperature and NG datasets. Table S1: Dysregulated miRNAs in samples stored at −20 °C, raw and normalized counts. Table S2: Kidney- RNAs raw and normalized counts for storage temperature dataset. Table S3: Kidney-RNAs raw and normalized counts for Isolation workflows.
