# Supplementary material for: Capturing the Kidney Transcriptome by Urinary Extracellular Vesicles—From Pre-Analytical Obstacles to Biomarker Research
Source: Genes (Basel). 2023 Jul 8;14(7):1415. doi: 10.3390/genes14071415 (PMC10379145; doi:10.3390/genes14071415)
Supplement: Supplementary file 1 [file genes-14-01415-s001.zip › SF_Barreiro et al.pdf]

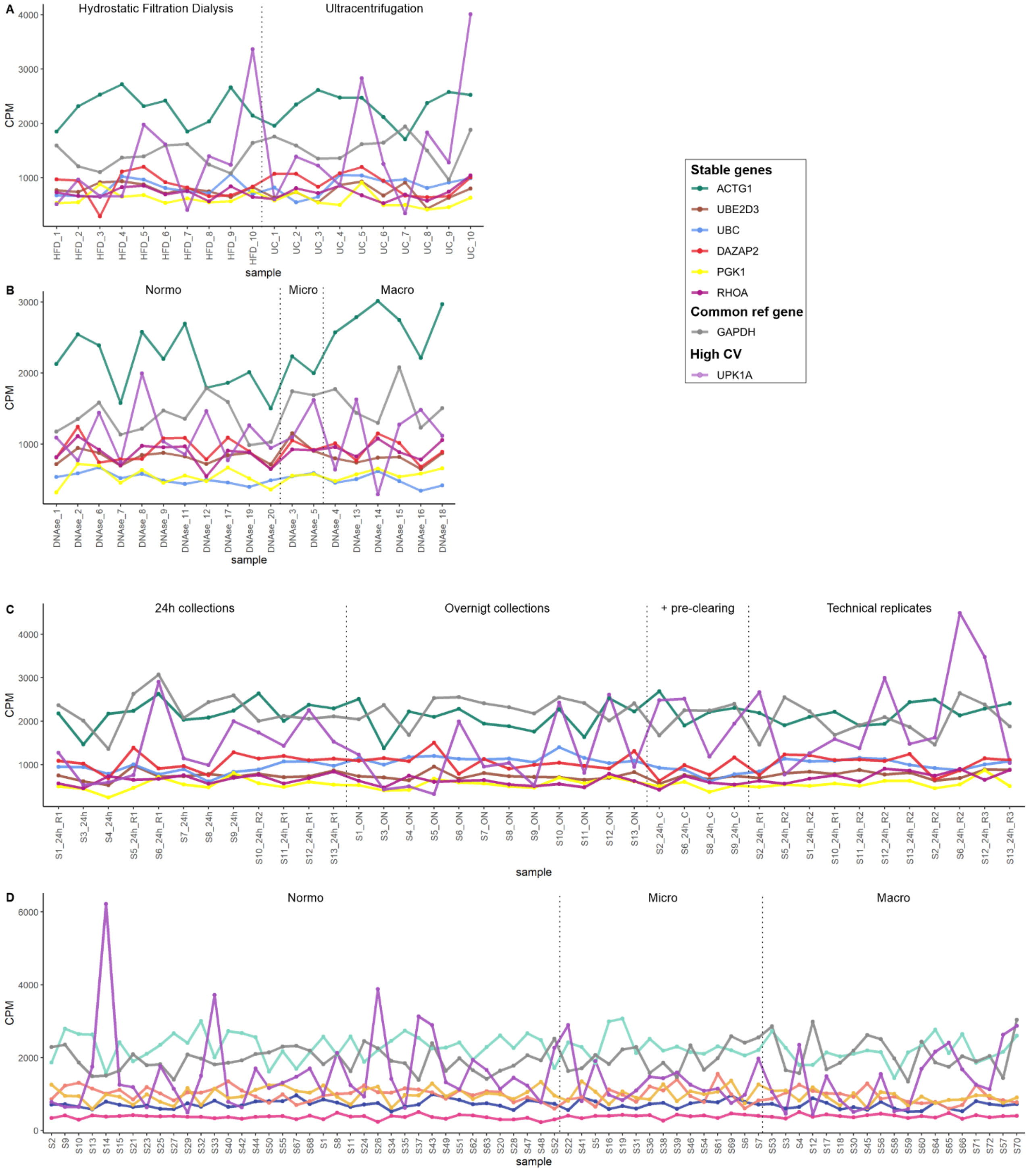

Figure S1: The mRNA sequencing read counts of six of the eleven candidate reference genes across samples in pre-analytical and DKD uEV datasets from men.

(A-D). Line graphs depicts CPM of ACTG1,UBE2D3,UBC, DAZAP2, PGK1and RHOA, across samples. A reference gene used commonly for normalization (GAPDH) and a gene with high CV in all datasets (UPK1A) were included.The uEV datasets included healthy controls and individuals with type 1 diabetes and different stages of albuminuria as well as comparisons of pre-analytical variables (all male). (**A).** EV isolation workflows, (**B).** In column DNAse treatment during uEV

RNA extraction, (**C**). A technical dataset (type of urine collection, pre-clearing the urine before freezing, and technical replicates) and (**D**). DKD cohort 1. Sample pairs or triplicates are named similarly apart from the abbreviation of the tested variable. Centrifuged (C), Coefficient of variance (CV), counts per million (CPM), hydrostatic filtration dialysis (HFD), macroalbuminuria (Macro), microalbuminuria (Micro), normoalbuminuria (Normo), ultracentrifugation (UC).

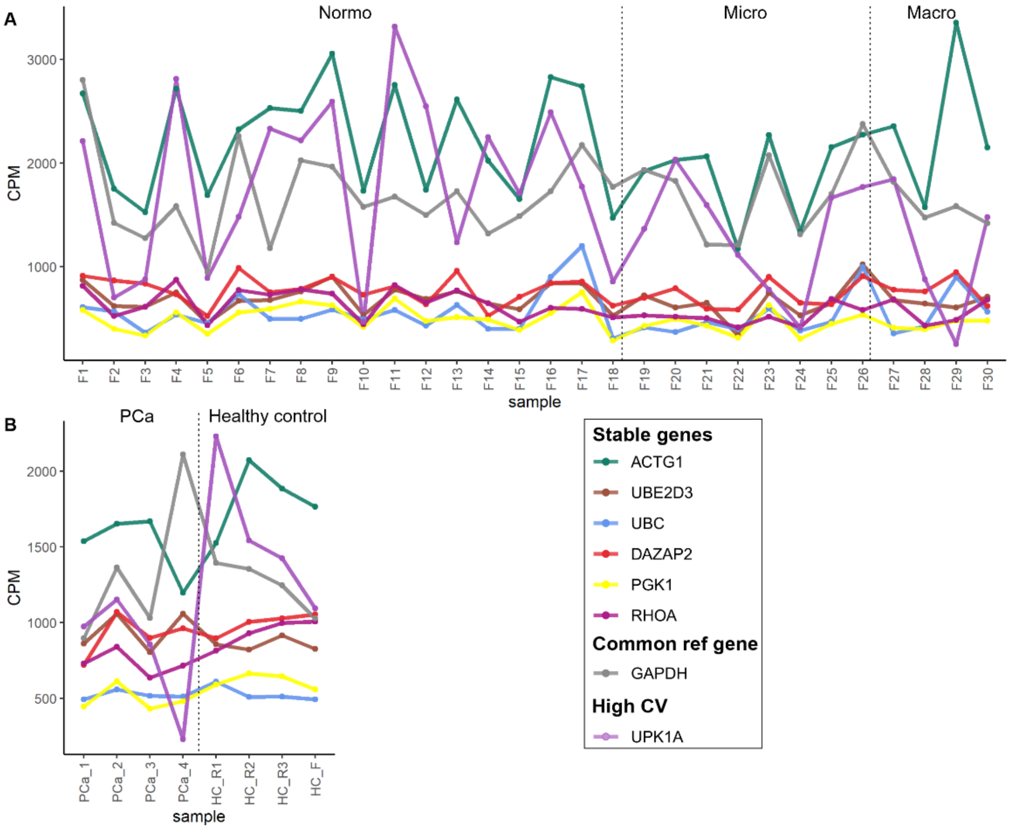

Figure S2: The mRNA sequencing read counts of six of the eleven candidate reference genes across samples in uEV datasets from DKD study of women and from prostate cancer patients.

(A,B). Line graphs depicts CPM of ACTG1,UBE2D3,UBC, DAZAP2, PGK1and RHOA, across samples. A reference gene used commonly for normalization (GAPDH) and a gene with high CV in all datasets (UPK1A) were included.The uEV datasets included (**A**). DKD cohort 2 (women with type 1 diabetes and different stages of albuminuria) and (**B**). PCa patients and healthy controls (technical replicates, R1-3). Samples PCa1, 3 and 4 were obtained before prostatectomy. Sample PCa2 was obtained after proctectomy from the same donor as PCa1. Coefficient of variation (CV), counts per million (CPM), macroalbuminuria (Macro), microalbuminuria (Micro), normoalbuminuria (Normo), and prostate cancer (PCa).

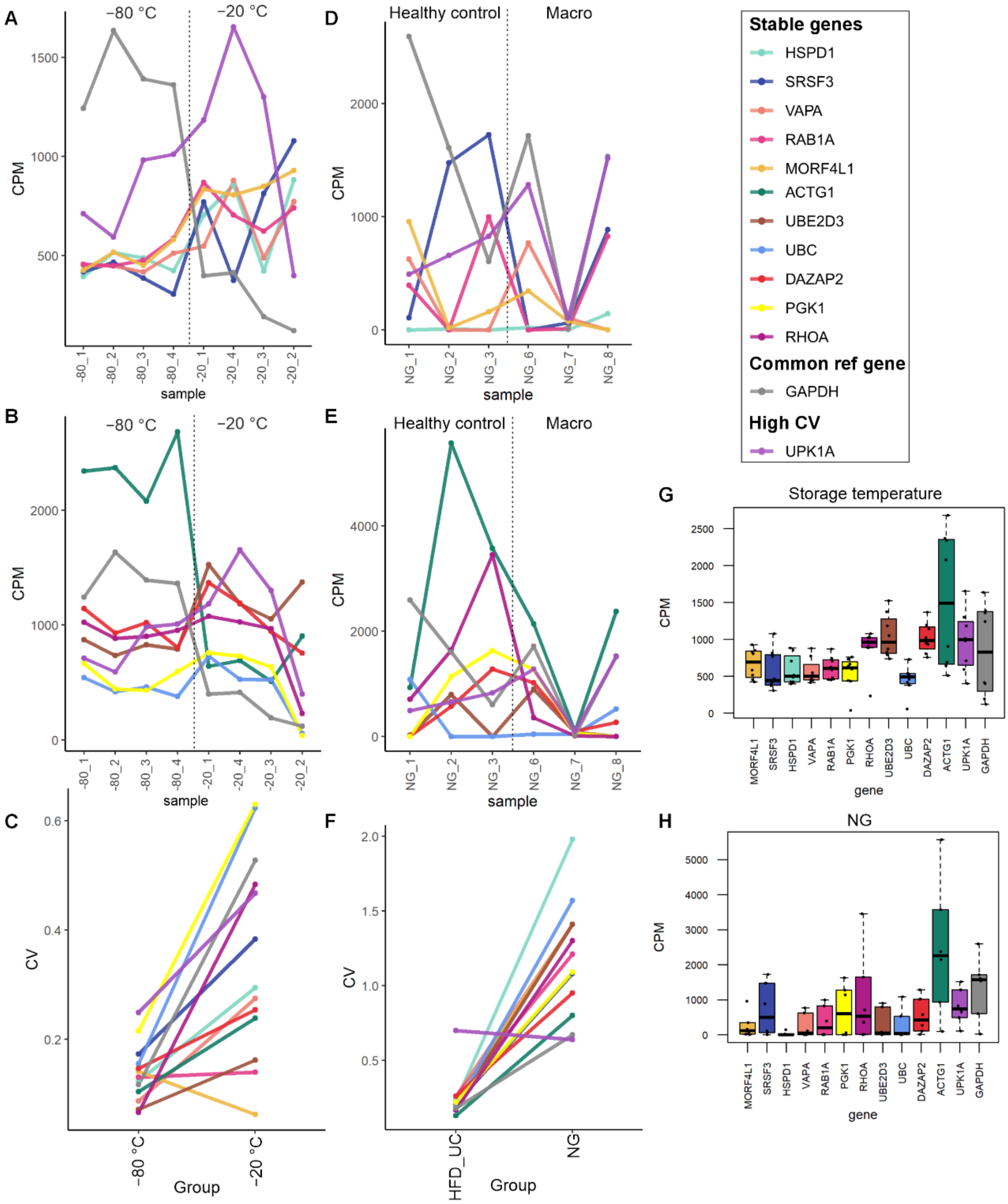

Figure S3: The mRNA sequencing read counts of candidate reference genes in storage temperature and NG datasets.

The uEV datasets included healthy controls and individuals with type 1 diabetes and different stages of albuminuria (all male). (**A,B and D,E)**. Line graphs depict CPM of candidate reference

genes across samples . A reference gene used commonly for normalization (GAPDH) and a gene with high CV in all datasets (UPK1A) were included. (**C,F**). line graphs depict the differences in CV for the candidate references between −80 and −20 samples and HFD, UC and NG samples. (**G,H).** Boxplots depict CPM per reference genes in storage temperature and NG datasets, respectively. Coefficient of variation (CV), counts per million (CPM), hydrostatic filtration dialysis (HFD), macroalbuminuria (Macro), Norgen urine Exosome Purification and RNA Isolation Midi Kit (NG). normoalbuminuria (Normo), ultracentrifugation (UC).
